# EMI-EDU: reframing learning and education through energy, mass and information

**DOI:** 10.3389/fnhum.2026.1761644

**Published:** 2026-08-07

**Authors:** Tommaso Firaux, Paola Damiani, Marco Cavaglià

**Affiliations:** 1 Department of Education and Human Sciences, University of Modena and Reggio Emilia, Modena, Italy; 2 Department of Mechanical and Aerospace Engineering (DIMEAS), Politecnico di Torino, Turin, Italy

**Keywords:** attractor dynamics, educational psychology, energy-mass-information (EMI) framework, evolutionary entropy, intersubjectivity and synchrony, learning processes, neuroplasticity, resonance and coherence

## Abstract

Building on the EMI framework that conceptualises consciousness, cognition, and behaviour as emergent phenomena arising from dynamic interactions among energy, mass, and information, this article extends the model into the educational domain by reframing learning as a resonance-based process within complex adaptive systems. While the initial formulation of EMI provided a scale-free biophysical account of mental phenomena, here we argue that neuroplasticity, evolutionary entropy, resonance and informational coherence constitute foundational principles for describing how minds learn, teach, and transform within relational and institutional environments. The proposed EMI-EDU perspective redefines the classroom as a dissipative field in which teachers and students operate as dynamic attractors, shaping shared cognitive and affective landscapes through synchronization, resonance, and phase transitions. Education is thus understood not as the linear transmission of content but as a systemic modulation of energetic-informational flows that stabilise new configurations of identity, competence, and relational agency. By integrating insights from neuroscience, physics, and pedagogy, EMI-EDU advances a pedagogy of resonance, plasticity, and systemic inclusion, offering a transdisciplinary framework capable of grounding inclusive practices in a scientifically coherent theory of mind and learning.

## Highlights

This article is part of a series of papers that articulate the Energy–Mass–Information (EMI) paradigm and its biophysical foundations. Each contribution is designed to be complementary:

- Unified Holographic Framework (UHF; Cavaglià and Tuszynski, 2026) introduces the biophysical substrate of the theory. It elaborates on the dual function of neuronal membranes, the role of coherent water domains, cerebrospinal fluid, and the coupling with large-scale electromagnetic phenomena. UHF provides both the mechanistic underpinnings and a portfolio of experimental strategies to test them.
- EMI Model (Firaux et al., 2026) develops a formalism and universal language for describing consciousness, cognition, and behaviour as emergent from dynamic transformations across energy, mass, and information. EMI is scale-free, allowing application from subcellular to systemic, interpersonal, and even societal levels. It establishes the conceptual and methodological criteria for falsifiability, empirical signatures, and integration with existing neuroscientific data.
- EMI–EDU (Firaux et al., 2026), the present paper, extends the EMI framework into the field of education and pedagogy. Here, learning is reconceptualised as a resonance-based process in which teachers and students co-create coherent informational fields. The classroom is reframed as a dissipative structure where attractor dynamics, plasticity, and systemic resonance drive the emergence of cognitive, emotional, and relational development.

## 1 Introduction

In recent decades, the traditional educational approach has entered a phase of epistemological crisis, urgently calling for models capable of integrating broader and more complex perspectives (Mason, 2008) to understand the human being in their entirety and to describe and improve teaching and learning processes, in the light of the new challenging scenarios.

The problem of consciousness, a fundamental subject in traditional philosophical and pedagogical reflection/speculation, has become one of the most ambitious goals of contemporary neuroscience which has been addressed by leading contemporary neuroscientists (Hunt et al., 2024). In general, as David Chalmers had already stated in 1996, the question of consciousness “can be approached in two ways: one simple, the other much more difficult” (Chalmers, 1996); recent scientific discoveries highlight the complicated relationship between brain, emotions, consciousness (LeDoux, 2000), opening up new challenges for the humanities and education and for a new inter-and trans-disciplinary dialogue.

In this scenario, recent advances in neuroscience and biology have proposed innovative models of brain functioning grounded in integrated biophysical principles, centred on the notions of holography and informational coherence. In particular, the UHF-EMI paradigm, developed by Cavaglià and Tuszynski (2026) and Firaux et al. (2026) has provided a transversal framework capable of describing cognitive and behavioural phenomena from the molecular to the systemic level, reconceptualising consciousness and learning as emergent phenomena arising from energetic, material, and informational interactions. Indeed, while current models can accurately describe certain aspects of human experience, they still lack comprehensive integrative capacity and often rely on reductive metaphors, such as the brain conceived merely as a predictive machine.

As Howard-Jones observed almost 20 years ago, “educational innovation involving valid neuroscientific concepts is a relatively new phenomenon and the challenges it poses are considerable, but it is reasonable to expect that progress in this field will accelerate with the growth of scientific understanding of the brain and mind” (Howard-Jones, 2008). Since then, research at the intersection of neuroscience and education has flourished, stimulating theoretical and applied inquiry on a transnational scale (Damiani et al., 2021).

Within this trajectory, the contributions of Frauenfelder and Santoianni (Frauenfelder and Santoianni, 1997; Frauenfelder et al., 2018; Frauenfelder, 1983) with the concept of bioeducation emphasised the co-evolution of biological and cultural dimensions of learning, while embodied cognition highlighted the central role of sensorimotor dynamics in shaping cognitive functions (Rizzolatti et al., 2001; Shapiro and Stolz, 2019; Wilson, 2002). These perspectives have progressively opened education to new epistemological horizons (Sibilio, 2021), underscoring the need for approaches able to overcome dualistic and reductionist interpretations.

Building on these advances, the EMI framework (Firaux et al., 2026) can be seen as a natural development that not only embraces the insights of embodied education (Gomez Paloma and Damiani, 2015; Damiani et al. 2021; Damiani et al., 2018; Francesconi and Tarozzi, 2012) but also provides them with a broader foundation. By interpreting learning as the emergent outcome of dynamic transformations among energy, mass, and information, EMI situates embodiment within a scale-free biophysical paradigm. Here, resonance, coherence, and attractor dynamics allow for a unified reading of educational processes that integrates neural, experiential, cultural, and relational dimensions.

In this sense, embodied education does not stand in contrast to EMI but rather finds within it a deeper confirmation: the body as mediator of cognition is understood as part of a wider architecture of distributed informational fields (Del Giudice et al., 1985; Levin and Martyniuk, 2018; Vitiello, 2001), in which learning emerges as a coherent reorganisation across multiple levels of reality.

This paper therefore emerges as a natural continuation of the UHF and EMI models, extending conceptual and applicative reflection to the educational context and providing theoretical and practical tools capable of complexly modelling neural and ontogenetic development. In summary, the aim is to promote a new pedagogy of resonance, plasticity, and systemic inclusion, which recognises and values the intrinsic complexity of the human subject and of contemporary educational environments.

In particular, considering the complex relationship between the brain, emotions and consciousness (Koch, 2004), we will focus on identifying a coherent mental model capable of supporting the “competences” to understand and intervene on aspects central to education/educational and teaching relationships, such as awareness and emotional skills, and, consequently, on training students and teachers in emotional-relational transversal skills.

Our hypothesis underlying the study and research we intend to pursue refers to the idea that a transdisciplinary redefinition, in line with current scientific models and the paradigm of complexity, underpinned by the EMI Model (Firaux et al., 2026) of the “extended mind” of both students (student/learner mind) and teachers (teacher mind) represents a conceptual enrichment and an essential condition for describing and understanding how it works and, consequently, for developing and implementing educational models capable of supporting the development of truly inclusive minds, such as empowering minds that generate learning/change and relational well-being.

## 2 The EMI model

### 2.1 Epistemological premises: how EMI-EDU should be read

Frameworks that span neuroscience, physics, and pedagogy face a specific epistemic hazard: the same word can simultaneously refer to a measured physical quantity, a falsifiable prediction, and a heuristic for practice. Without an explicit articulation of which use is at stake in each passage, readers are forced to either over-interpret the model as a reductive physicalism or to dismiss it as evocative metaphor. We therefore distinguish at the outset three distinct registers in which the framework operates.

Imported substrates. Several mechanisms invoked in the paper, phase-locking in inter-brain hyperscanning data (Liu et al., 2018; Nam et al., 2020), heart-rate variability coupling between interacting individuals (McCraty, 2022), metastable attractor dynamics (Cabral et al., 2022; Deco et al., 2017; Tognoli and Kelso, 2014), neural oscillations as carriers of coordinated processing (Buzsáki and Draguhn, 2004), are not posited *de novo*. They are imported from established peer-reviewed work as empirical anchors. The framework’s task is to articulate how these mechanisms relate to educational processes, not to redefine them (Table 1).

**Table 1:** Core EMI–EDU principles and illustrative empirical operationalisations.

EMI–EDU principle	Educational domain	What it describes	Illustrative empirical measures
Evolutionary entropy	Developmental and learning trajectories	Directionality and complexity of learners’ cognitive, affective and identity change over time	Longitudinal analyses of task complexity, concept maps, portfolio work; variability and convergence in performance indices
Pedagogical resonance	Teacher–student and peer interaction	Degree of synchronisation among brains, bodies and behaviours during educational activities	Hyperscanning EEG/fNIRS; heart rate variability (HRV) synchrony; behavioural synchrony (movement, gaze, speech timing)
Temporal & rhythmic design	Lesson structure and classroom management	How instructional time, transitions, pauses and cycles are organised to support metastable, plastic states	Coding of lesson phases and transitions; temporal patterns of engagement; momentary self-report of attention and fatigue
Multimodal embodied synchronisation	Embodied activity in the classroom	Co-movement, gesture, posture, vocal prosody and gaze that align learners and teachers	Video-based movement analysis; motion capture; prosodic analysis; synchrony indices for gesture and posture
Affective & autonomic co-regulation	Emotional climate and regulation of arousal	Joint regulation of affect and autonomic states enabling safety, exploration and reflective functioning	HRV and other autonomic measures; self- and other-report of affect; observational scales of classroom emotional climate
Pointer & environment design	Physical, symbolic and institutional environment	Configuration of space, artefacts, symbols, rituals and rules that guide trajectories in the educational state space	Qualitative and quantitative assessment of classroom layout and artefacts; analysis of rules/rituals; inclusion and participation indices

Generated predictions. EMI-EDU is testable to the extent that it formulates predictions that would not be drawn from existing pedagogical or neuroeducational frameworks. These predictions are listed in Table 2 and concern measurable changes in inter-brain synchrony, autonomic coupling, behavioural coordination, and longitudinal trajectories of cognitive complexity, conditional on specific pedagogical interventions. The predictions are stated with directions, expected effect sizes anchored to existing benchmark literature, and falsification conditions.

**Table 2:** Falsifiable predictions generated by the EMI-EDU framework.

ID	Construct	Experimental design	Primary metric	Expected direction and benchmark	Falsification condition
P1	Pedagogical resonance	Within-subjects comparison of EEG hyperscanning during (i) traditional frontal lecture and (ii) sense–tune–stabilise structured collaborative session, matched for content and duration	Inter-brain Phase-Locking Value (PLV) in theta (4–7 Hz) and alpha (8–13 Hz) bands across teacher–student and student–student dyads	PLV during structured collaborative sessions significantly higher than during frontal lectures; expected effect size d ≥ 0.4 in theta band, consistent with classroom hyperscanning benchmarks (Dikker et al., 2017; Bevilacqua et al., 2019)	Absence of significant PLV difference (or reversed direction) at p < 0.05 with adequately powered sample (n ≥ 12 dyads)
P2	Affective and autonomic co-regulation	Comparison of two structurally matched group sessions (same content, same duration) differing only in the explicit application of co-regulation strategies (validation of emotional states, prosodic modulation, narrative framing) versus standard delivery	Cross-participant heart-rate variability (HRV) synchrony, computed via cross-correlation of RMSSD or HF-HRV time series	Significantly higher HRV synchrony in co-regulated condition; expected cross-participant r ≥ 0.3, consistent with dyadic co-regulation literature (Suveg et al., 2016; McCraty, 2022)	Equivalent or lower HRV synchrony in co-regulated condition
P3	Phase transitions and attractor reconfigurations	EEG recording during collaborative problem-solving tasks with self-reported insight events (“Aha! moments”) timestamped by participants	Metastability indices (Kuramoto order parameter variability, Lempel–Ziv complexity, EEG microstate transition probability) in the 5–10 s preceding self-reported insights vs. matched control intervals	Pre-insight intervals show significantly higher metastability and increased microstate transitions, consistent with dynamic flexibility findings (Deco et al., 2017; Cabral et al., 2022; Tognoli and Kelso, 2014; Sandsten et al., 2020)	Absence of metastability modulation around insight events, or modulation in the opposite direction
P4	Pointer and environment design	Between-classroom or within-classroom counterbalanced manipulation of physical layout (circular vs. frontal seating) during equivalent collaborative tasks	Turn-taking entropy (Shannon entropy of speaking-turn distribution) and gaze-distribution entropy from video analysis	Circular seating produces significantly higher turn-taking and gaze entropy (more equitable distribution), consistent with classroom interaction research (Marx et al., 2000; Rogers, 2020)	Equivalent entropy distributions across conditions

Each row specifies the EMI-EDU construct, the experimental design that operationalises it, the primary metric, the predicted direction with anchored benchmarks, and the empirical condition that would falsify the prediction.

Heuristic constructs. Some elements of the framework, the classroom as a resonant field, the sense–tune–stabilise protocol, pointer design, operate not as mechanistic claims but as organising categories for pedagogical practice. Their function is to render educators perceptually and operationally sensitive to multiscale dynamics that conventional frameworks tend to leave implicit. They are accountable to the empirical predictions of the second register, but they are not themselves hypotheses to be tested in isolation.

In what follows, when we use terms such as “resonance,” “coherence,” or “field,” we indicate which register is operative. The transfer of physical vocabulary into educational discourse is therefore not analogical in the loose sense, nor reductive in the strong sense. It is dimensioned: energy, mass, and information have distinct operational referents at the neural scale (metabolic and oscillatory dynamics, neuronal substrate, synaptic encoding) and at the relational scale (cognitive effort, embodied co-presence, communicative content), and the framework’s central commitment is to specify how these scales couple, not to collapse them into one another.

### 2.2 Premises: levels and principles

The EMI model takes as its biophysical substrate the body of work developed within the Unified Holographic Framework (UHF; Cavaglià and Tuszynski, 2026), which articulates a ‘Dual Function’ role of neuronal membranes as both electrochemical and computational structures. UHF brings together evidence on lipid-mediated membrane organisation (Lingwood and Simons, 2010), structured water domains and their electrodynamic properties (Pollack, 2001), and the role of phospholipid composition in cellular coherence (Vernuccio et al., 2025), within a broader account of brain function as a multiscale informational architecture. The present paper imports these substrates rather than re-derives them: when EMI-EDU speaks of coherence, it refers to a convergent pattern of informational integration across the substrates documented by UHF and across the neural oscillatory regimes that depend on them (Buzsáki and Draguhn, 2004), enabling the emergence of stable cognitive and behavioural states (Firaux et al., 2026).

The EMI paradigm (Energy-Mass-Information; Firaux et al., 2026), provides an innovative lens for interpreting cognitive and behavioural phenomena as the result of continuous dynamic transformations among three interdependent domains:

Energy: Encompasses all oscillatory dynamics and electromagnetic variations within the system, including ionic currents, photon emissions (UPE), electromagnetic field fluctuations, and thermodynamic gradients. It also includes the energetic dynamics of other systems with which the individual interacts.

Mass: Refers to the physical substrates that materially support cognition, such as lipid neuronal membranes, structured water, microtubules, and cerebrospinal fluid (CSF), which are crucial mediators in the holographic encoding of information. It also includes the mass of interacting systems.

Information: Constitutes the set of coherent field configurations, phase-aligned neural oscillations, symbolic codings, and patterns of energetic interference underlying subjective content and emerging cognitive meanings. Being distributed, information is not confined to individual dynamics but co-constructed through interaction with other elements and systems.

### 2.3 Levels of the EMI model

Subcellular. Subcellular EMI dynamics emerge from interactions among lipid-organised membrane domains (Lingwood and Simons, 2010), structured water with its exclusion-zone properties (Pollack, 2001, 2013), local electromagnetic fields generated by membrane and cytoskeletal components, and ephaptic coupling among neighbouring neurons. These mechanisms, developed in detail within the UHF framework (Cavaglià and Tuszynski, 2026; Cavaglià et al., 2023), are imported here as candidate channels for the transduction of information between scales; their photoelectric and piezoelectric properties are not further developed in the present paper but provide the substrate-level continuity between EMI-EDU and the broader EMI–UHF programme.

Cellular. EMI coherence emerges from the integration of action potentials, membrane oscillations, and cell-to-cell synergies. Membrane potential, nested rhythms, and cooperation via cerebrospinal fluid ensure the informational coherence and cellular plasticity essential for learning and memory (Cavaglià and Tuszynski, 2026; Llinas, 2002).

Systemic. At the systemic level, local coherent oscillations aggregate to create macroscopic electromagnetic fields (Buzsáki et al., 2013; Buzsáki and Draguhn, 2004). Cognitive states correspond to stable configurations (attractors; Cabral et al., 2022; Deco et al., 2017) in a dynamic and chaotic field, enabling flexible and adaptive transitions, and illustrating how neural plasticity and systemic coherence guide learning processes (Firaux et al., 2026).

Environment. Environmental dynamics, in the EMI framework, comprise the coupling of individual organisms with their physical, social, and informational surroundings. The UHF framework (Cavaglià and Tuszynski, 2026) explores in detail the role of large-scale electromagnetic couplings between organisms and their planetary environment as candidate substrates of this interaction. In the present paper, we restrict the educational application to interpersonal and group-level forms of environmental coupling, particularly interpersonal synchronization (Carollo et al., 2021; Deng et al., 2023; Gaggioli et al., 2019; Stupacher et al., 2017) and the shared phenomenological field generated by group dynamics (Gordon, 2025; Kavaliauskaitė et al., 2024; Marzoratti and Evans, 2022; Nam et al., 2020; Yang et al., 2022; Yuan et al., 2022), which provide measurable channels for empirical investigation in classroom settings. These dynamics underscore the role of the educational environment as a resonant and modulatory space for learning.

### 2.4 Attractors and neuroplasticity

Neuroplasticity exemplifies the core logic of the EMI model, illustrating how learning arises from the dynamic interaction of energy (neural oscillations and metabolic flows), mass (synaptic structures and membrane architectures), and information (symbolic encoding and experiential patterns; Firaux et al., 2026). Each cognitive state corresponds to a stable attractor within a chaotic field, and phase transitions-such as those observed during critical learning periods-mark the system’s shift from one attractor landscape to another (Kozma et al., 2021; Scott Kelso, 2012). These transitions enable flexible reconfigurations without loss of global coherence. Within the EMI framework, neuroplasticity is understood as the material imprinting of information through energetic processes: repeated, resonant experiences stabilise energetic-informational configurations, guiding the system’s evolution over time (Brennan et al., 2023; Miller, 2016). Thus, each act of learning reflects the emergence or consolidation of an attractor, embedding meaning within the brain’s electromagnetic and structural landscape (Cabral et al., 2022; Deco et al., 2017; Galadí et al., 2021).

Neuroplasticity thus stands as a paradigmatic example of how information becomes materially inscribed through energetic transformation-capturing the intimate interplay between the three core dimensions of the EMI framework: energy, mass, and information. From the perspective of complex systems physics, learning can be described as a phase transition, occurring when the neural system crosses a critical threshold of stimulation and reorganises its internal dynamics (Scott Kelso, 2012). These reorganisations are governed by nonlinear processes and by the interplay between excitation/inhibition mechanisms, resonance, and synaptic modulation (Firaux et al., 2026; Cavaglià and Tuszynski, 2026).

Within EMI, plasticity is not a purely random or mechanical adaptation but the result of entropic modulation: the capacity of the neural system to explore thermodynamical landscapes through the selection and stabilisation of meaningful patterns (Firaux et al., 2026). Demetrius’s notion of evolutionary entropy (Demetrius, 2023; Demetrius, 2024), introduced more fully in §3.1 in its educational application operates here as a structural invariant: it describes the rate at which a system transforms fluctuating inputs into energy-efficient configurations and, applied to neural dynamics, characterises the conditions under which new cognitive attractors emerge, stabilise, or dissolve. The directionality implied by this measure is not teleological but emerges from the internal logic of the system and its dynamic coupling with environmental inputs.

## 3 Toward an EMI model in education (EMI-EDU)

Starting from the theoretical premises of the EMI Model outlined above, we will now attempt to explore principles and elements for an initial application of the EMI Model to Education, with particular attention to the definition of a coherent model of mind.

The EMI model frames mind and consciousness as scale-free dimensions-phenomena that cannot be reduced to a single dynamic level. The model describes the mind as a coherent field in which complex landscapes of attractors operate in conjunction. Due to the nonlinear nature of mental and conscious phenomena, attractors are defined as equilibrium points in which a chaotic system stabilises for a given period, under specific conditions relating to its energy, mass, and information. In EMI, the self is viewed as the system’s principal attractor-structured over time and simultaneously shaping and being shaped by the individual’s lived experience. Each attractor has well-defined bioenergetic constraints.

To clarify how fundamental this dynamic is in educational contexts - and how every human phenomenon can be described through physical laws - we must highlight key mechanisms underlying brain functioning.

EMI reformulates phenomena such as consciousness, cognition, emotion, and behaviour within the dynamics of attractors. Every act is described as an energy dynamic that writes information onto a mass, allowing information to stabilise and manifest in matter. Landauer’s principle states that every such process entails a cost. While this principle originates in digital information theory, biological information incurs a much higher energy cost (Craddock et al., 2009; Sartori et al., 2014; Street, 2020). Based on this, we can assert that every thought or emotion has a physical cost, and we can begin to study them as measurable physical phenomena-no longer as merely subjective or intangible dimensions. EMI proposes that every complex cognitive state-including its emotional and behavioural components-is the result of the subject’s energetic state, which is itself shaped by the guiding attractor (Firaux et al., 2026). To “disrupt” or shift an attractor, a critical perturbation is needed - either through a significant external event or through the accumulation of minor efforts that push the system toward a critical point and phase transition toward a new attractor (Deco et al., 2017; Galadí et al., 2021; Kozma et al., 2021; Scott Kelso, 2012; Tewarie et al., 2025).

Let us consider a few examples. In the context of learning, each new piece of information entails costs associated with its search, internalisation, assimilation, and subsequent expression. However, this learning process is not linear - it involves the whole attractor system. New information introduces the organism to a novel energetic imprint, altering it from the very moment of engagement. In this sense, exposure to new information already implies internal changes and energetic costs. Once a skill is acquired in a specific domain, it affects the entire dynamic landscape (to varying degrees depending on context) and leads to evolution in other areas as well.

Concrete examples can be drawn from studies on 4E cognition (Garbarini and Adenzato, 2004; Newen et al., 2018), placed at the foundation of the Embodied Education, which show that emotional-relational, cognitive, social, and educational dimensions are tightly interconnected (Damiani et al., 2021).

In a 2020 study, Damiani and Gomez Paloma highlighted the existence of some “bridge dimensions” or transdisciplinary dimension between neuroscience, psychoanalysis, and pedagogy, enhancing their mutual fertilisation within an inclusive and systemic framework (Damiani and Gomez Paloma, 2020). This perspective, already outlined in the psychotherapeutic and neuroscientific fields by authors such as Mark Solms (Solms, 2000) and Mauro Mancia (Mancia, 1999), encourages a radical rethinking also with regard to didactic action and the design of the school curriculum, in which educational processes are not limited to the transmission of information but are conceived as opportunities for integral development, in harmony with the principles of complexity and interconnection.

These dimensions are fundamental for development and learning processes and also play a key role in understanding neurodevelopmental disorders as well as the functioning of the *extended mind* in general. Among the *bridge dimensions* identified: Bodily Ego and Cognitive Unconscious (Implicit Mental System); Empathy; Intersubjectivity; Emotional Functions and Vital Forms (Stern, 2010).

By integrating the perspectives of study on Bridge dimensions and, more generally, the framework of Embodied Education with the EMI paradigm, we thus propose a view of education as a distributed energetic-informational phenomenon, wherein learning entails the stabilisation of cognitive and behavioural attractors through dynamics of resonance, rhythm, and coherence.

This approach redefines the school as an open and dissipative system, capable of self-organisation and continuous transformation, where the teacher assumes the role of facilitator of systemic coherence, and students are regarded as active nodes within a shared phenomenological field.

In summary, the aim is to promote a new pedagogy of resonance, plasticity, and systemic inclusion, which recognises and values the intrinsic complexity of the human subject and of contemporary educational environments.

Additionally, attachment research reveals that parenting style-i.e., the energetic-informational resources to which one is exposed-predicts developmental trajectories (Adenzato et al., 2019; Carbone et al., 2025; Carbone et al., 2024). This has important implications for our model, as it is located in the open and dynamic space of border and exchange between life science and human science and contributes to enriching the reflection on the transdisciplinary “*bridge dimensions*,” mentioned above, in an updated perspective, based on scientific evidence, for their concrete operationalization.

Central to this is Demetrius’ theory, which analyses how the resources available to a system determine its trajectory based on its level of evolutionary entropy, i.e., the degree of cooperation among nodes in a complex system (Demetrius, 2023). The theory of directionality, emerging from this model, posits that a system, given stable access to a certain level of resources, tends to evolve toward greater complexity and self-organisation. This model can therefore be applied to learning contexts. Foundational information, viewed as essential resources, enables the subject to organise themselves in specific ways and pursue the most evolved developmental trajectory in alignment with personal goals and intentions.

Complex, deep, extensive educational relationships can be described as *evidence-based* foundational elements (generating shared biophysical fields) that explain and make possible subjectivity and agency within a model of extended ecological-relational intersubjectivity (mind–body–environment).

We use the term *relational agency* to denote the capacity of a subject to act and self-organise within, and through, the trans-individual dynamics of the resonant field, rather than as an isolated agent. The construct is related to but distinct from Edwards’s notion of relational agency in collaborative work (Edwards, 2005), Trevarthen’s primary intersubjectivity, Gallese’s embodied intersubjectivity grounded in shared sensorimotor representations (Gallese, 2003, 2014), and Bandura’s social agency: it specifies that agency emerges from the dynamic coupling between the individual’s biophysical state and the multiscale field in which the individual is embedded, and is therefore measurable through indices of inter-brain synchrony, autonomic coupling, and behavioural co-regulation rather than through individual-level metrics alone.

The Energy-Mass-Information dynamics provide a biophysical explanation for neuroplasticity and for how learning can modify the brain’s biophysical structure. Learning is thus grounded in neuroplasticity.

The individual is no longer seen as a linear computer but as a sophisticated and complex organism functioning as an antenna, transducing signals within the unified field. This radically alters the educational paradigm. It returns us to a truly *educational* dimension-*educere*-where the goal is to draw out the full potential originally present within the person. The emergence of this potential is tightly linked to the individual’s tuning with the surrounding field. Learning, therefore, means learning how to extract information from the field and render it coherent within one’s system. Different environments possess different fields, which in turn influence the emergence of different ideas, depending on the informational-energetic field in which one is immersed. Moreover, learning is a continuous process: every interaction we have with our environment implies, to some degree, a modification of the attractor that constitutes our identity. Education plays a fundamental role here-in shaping individual identity.

In our model, identity is bound to all the system’s energy, mass, and information dynamics, and is thus observable across multiple biophysical dimensions, as previously outlined. Learning to build one’s identity and to attune to one’s inner being is, therefore, the most essential form of education. Self-awareness-and, consequently, connectedness with oneself, others, and the field-takes on a concrete biophysical measurability.

Within EMI, one of the foundational paradigms is constructivism (Guidano, 1991; Guidano, 1987). Learning does not merely mean absorbing bits of information, but rather knowing how to construct a reality that is most coherent with one’s deepest nature. The construction of reality is tied to the bioenergetic landscape described by EMI. More resources-energetic, emotional, relational, linguistic, cognitive, etc.- correspond to the ability to access a wider range of trajectories from which to consciously choose one’s evolutionary path. Furthermore, one’s biophysical state actively influences the environment, contributing to the evolution of the complex system in which we are embedded and of which we are an integral part. Just as neurons are nodes within the brain’s complex system, we are nodes within a broader reality, one that we both influence and are simultaneously influenced by.

Similarly, the school can be viewed as a semi-open thermodynamic system, in which teachers play a fundamental role in shaping the equilibrium of the complex system (Davidesco, 2020; Hollan, 2020). Starting from individual students, moving through classes, year levels, academic tracks, and reaching the entire institution and inter-institutional networks, it is possible to observe complex dynamics through a biophysical lens that, for the first time, unifies levels from the micro to the macro scale.

### 3.1 Evolutionary entropy and the structure of cognitive dynamics

Building on the formal characterisation introduced in §2.4, this section develops the educational application of evolutionary entropy. A clarification is in order at the outset: Demetrius’s notion of evolutionary entropy (Demetrius, 2023; Demetrius, 2024) is not equivalent to Boltzmann’s thermodynamic entropy or to Shannon’s informational entropy. It is a population-level measure originally developed to quantify the rate at which a biological system transforms fluctuating resources into stable, energy-efficient configurations through cooperation among nodes—mathematically, it is related to the variance of the demographic generation cycle of a system. When applied to neural and cognitive dynamics, evolutionary entropy characterises the system’s capacity to convert variable inputs into robust, integrated patterns: high evolutionary entropy corresponds to configurations in which subsystems cooperate efficiently in stabilising shared informational regimes (Sarasso et al., 2024).

Within EMI-EDU, this measure provides a principled criterion for evaluating educational configurations. Cognitive development can be understood as a directional trajectory through configurations of varying evolutionary entropy: each phase of learning corresponds to a transition in which the system reorganises its attractor landscape toward greater integration and adaptive robustness. This directionality is not linear nor teleological; it is emergent, shaped by the internal logic of the system and its dynamic coupling with environmental inputs.

The educational implication is that pedagogical configurations characterised by structured cooperation, varied environmental pointers, and rhythmically organised transitions tend to support trajectories of higher evolutionary entropy, whereas rigidly competitive or impoverished configurations tend to lower it. This connects evolutionary entropy directly to the operational levers articulated in §5.2 and to the longitudinal predictions discussed in §6.

### 3.2 Pedagogical resonance

Within the EMI framework, resonance describes the emergence of coherence across energetic and informational domains, enabling systems to stabilise and reorganise through rhythmic synchronization (Hunt and Schooler, 2019; Jones and Hunt, 2023; Young et al., 2022). Translating this principle into the educational field, we propose the notion of *pedagogical resonance*, understood as the capacity of teachers and students to enter a shared oscillatory field in which cognition, emotion, and behaviour align dynamically. Learning, in this perspective, is not the mechanical acquisition of isolated contents but the stabilisation of resonant patterns that connect individual and collective minds through rhythms of attention, gesture, language, and affect. Interpersonal synchrony has been documented across speech, social interaction, and intimate dyads (Amiriparian et al., 2019; Hu et al., 2022; Dewitte, 2024).

Theoretically, pedagogical resonance can be described as the dynamic coupling of attractors within a complex system: each cognitive or affective state corresponds to a local attractor, and resonance emerges when different nodes of the system-teacher and students-achieve phase alignment, generating a coherent attractor landscape. This dynamic is not merely conceptual but can be empirically investigated through multiple measurement channels already outlined in the EMI framework (Hunt et al., 2024). At the neural level, hyperscanning EEG allows the detection of inter-brain phase-locking and cross-frequency coupling (Liu et al., 2018; Nam et al., 2020), while magnetoencephalography (MEG) and near-infrared spectroscopy (fNIRS) provide complementary measures of large-scale synchronization (Lu et al., 2023; Zhang et al., 2024). At the physiological level, indices such as heart rate variability (HRV) (McCraty, 2022), respiratory coupling, galvanic skin response, and pupillometry capture fine-grained oscillatory alignments that reflect shared states of arousal and regulation. At the level of broader bioelectromagnetic dynamics, ultra-weak photon emissions (UPE) and electromagnetic field recordings, investigated in depth within UHF (Cavaglià and Tuszynski, 2026; Zarkeshian et al., 2022), represent exploratory channels for assessing systemic coherence that may underlie collective attractor states. We mention them here as part of the broader EMI–UHF substrate, while noting that their direct operationalisation in educational settings remains an open question for future research.

Pedagogical resonance, therefore, grounds itself in a measurable substrate of energetic–informational dynamics, bridging the physics of oscillatory coherence with the phenomenology of teaching and learning. The most transformative educational experiences are those capable of inducing phase transitions in the shared attractor landscape, where resonance facilitates the stabilisation of new cognitive, affective, and relational configurations. In this view, the classroom can be described as a resonant field in which knowledge, identity, and agency emerge through the dynamic entrainment of minds, bodies, and environments.

## 4 The educational environment as a field

Within the EMI model, intersubjectivity is a foundational and irreversible dynamic of the system. It is impossible not to be in relation with something, and the exchange between parts becomes essential. Moreover, the very concept of “parts” is a construct arising from the rational mind’s tendency to individuate and generate recursive patterns.

Education begins with the ability to relate to the other. In bio-physical terms, this capacity can be described as the ability to regulate one’s own energy-informational state in order to enable synchronization between different individuals and the emergence of new meanings and configurations (Pan et al., 2021; Wlodarczyk et al., 2020; Yan et al., 2025). Relationship is thus understood as a multi-level energy-informational exchange that gives rise to a third element-the relationship itself-which is more than the sum of its parts. This dynamic is present both in dyads and in larger groups, and it manifests in increasingly sophisticated forms while adhering to the same underlying principles. There is, therefore, a directionality-not in the sense of a predetermined goal, but as an evolutionary trajectory that converges toward a new attractor, in a nearly infinite fractal dynamic.

The Relationship, consistently with pedagogical and psychodynamic perspectives, is described as a contextual and processual element. However, within the EMI framework, the understanding of certain dynamics and essential aspects-emerging from exchanges among other contextual elements at different levels-is expanded. Accordingly:

1. the relationship can be described, observed, and modified from multiple vantage points, ranging from the micro to the macro level and with varying degrees of depth;
2. every change in/on the relationship implies a change in the other elements (students, teachers, class) and vice versa.

We can therefore state that one added value of adopting the EMI perspective in education lies in the possibility of an “extended understanding” of this key object-the educational and didactic relationship-according to a scientifically grounded, complex, and integrated perspective. This framework broadens the essential (constitutive) explanatory factors that generate the relationship and, consequently-though not in a linear way-enriches the interpretative, observational, and intervention pathways concerning it.

As Firaux et al. (2026) argue, rituals, shared experiences, language, forms, and colours generate a sense of identity that arises from synchronization with a given wave phase. For example, a group that shares specific words, habits, colours, sounds, or behaviours tends to recreate the same experiences-resulting from the interaction between their physical bodies and the environment or field to which they are connected (Dikker et al., 2017; Li et al., 2024; Liu et al., 2021). These principles, closely aligned with 4E cognition (Newen et al., 2018), determine the emergence of shared attractors and meanings that produce bio-physical realities-either converging toward similar outcomes or diverging, depending on the interaction of differing energy-informational states.

In other words, although an attractor tends to unify the shared experience and draw it toward a centre, personal attractors may produce different results due to the unique informational configurations they embody. The same event, in this view, generates different reactions in different individuals.

The classic axiom “it is impossible not to communicate” can be reformulated here as “it is impossible not to interact.” Education, in all its complexity-composed of places, languages, relationships, emotions, and rituals-actively contributes to the formation of the subject’s identity. The construction of identity, along with the development of a biological system that grows and evolves, are critical processes. Having clear guidelines for the intentional structuring of one’s mental, emotional, and behavioural experience becomes of utmost importance. If every conscious, cognitive, and behavioural act is supported by a physical substrate, then knowing how to manage one’s own thinking-creative energy par excellence-becomes a vital educational goal. If, as previously argued, we shift paradigms from viewing the brain as a computer to viewing it as a tuned radio, then education can no longer be conceived as the mere filling of the student’s head with sterile information. It must instead include self-knowledge as a foundational component and tuning as a general principle.

Complex, deep, and extended relationships-considered as foundational, evidence-based elements (generating shared biophysical fields)-explain and make possible subjectivity and agency within an ecological–relational model of intersubjectivity (mind, body, environment), consistent with the embodied education framework.

## 5 EMI–EDU as a resonance-inducing educational model

The previous sections have framed learning and development within the EMI paradigm, highlighting how cognitive, affective, and relational trajectories can be understood as movements in an attractor landscape shaped by energy mass information dynamics. EMI EDU now takes a further step: it does not only *describe* educational phenomena in these terms, but it offers a normative and operational model for deliberately inducing coherence and resonance in educational contexts.

In this perspective, the classroom is conceived as a tunable resonant field. Teachers, learners, and the wider institutional setting become components of a multi-level system whose configurations can be modulated through specific pedagogical choices. Coherence and resonance are therefore not simply emergent properties to be observed, but design variables that can be cultivated, monitored, and stabilised over time.

### 5.1 Classroom as a tunable resonant field

Within EMI-EDU, every educational setting is described as a semi-open, non-linear system that constantly exchanges energy, matter, and information with its environment. At the micro level, neural and bodily processes embody these exchanges; at the meso level, interpersonal dynamics and group processes give them a relational form; at the macro level, institutional and cultural structures constrain and guide the possible trajectories of the system.

The key assumption is that these levels are coupled through resonance mechanisms: rhythmic alignment, phase-locking, and synchronisation across multiple scales (neural oscillations, autonomic rhythms, motor patterns, conversational turn-taking, ritualised practices). Pedagogically, *resonance* names a specific relational quality between learner, teacher, and content, anticipated by dialogic approaches, by Csíkszentmihályi’s notion of flow (Csíkszentmihályi, 1990), and by Vygotsky’s zone of proximal development. These frameworks describe what resonance looks like in educational practice; EMI-EDU contributes by specifying the conditions under which it arises and the mechanisms through which it operates. Within this view, resonance is the relational state that makes cognitive *phase transitions* possible: while the phase transition is the developmental outcome, a reconfiguration of the learner’s attractor landscape, resonance is the energetic and relational condition that allows the leap to occur. A classroom is thus a resonant field whose configuration at any given time reflects a specific pattern of coherence and decoherence among its components.

EMI-EDU defines the educational task as the intentional modulation of this field. Rather than acting only on isolated variables (content, individual performance, standardised outcomes), the model proposes that educators should learn to *sense, tune, and stabilise* the global configuration of the field, using coherence and resonance as guiding principles for design and intervention.

### 5.2 Operational levers for inducing coherence

To translate this idea into practice, EMI–EDU identifies a set of operational levers through which teachers and institutions can actively shape the resonant properties of the educational field. At least four domains are particularly relevant:

1. Temporal and rhythmic design
  Educational activities always unfold in time. EMI–EDU emphasises the role of temporal structuring, the way lessons begin and end, how transitions are managed, how pauses and intensifications are distributed. Recurring cycles, micro-rituals, and carefully tuned temporal patterns can move the system away from chronic hyper- or hypo-arousal and toward metastable regimes, in which variability and order are optimally balanced. In such regimes, neuroplasticity and exploration are facilitated, and the probability of constructive attractor transitions increases.
2. Multimodal embodied synchronisation
  Human interaction is fundamentally embodied. The model therefore assigns a central role to practices that foster multimodal synchronisation between teachers and students: coordinated movement, shared gaze, postural mirroring, prosodic alignment, choral speech, collective creative actions. These activities promote interpersonal neural and physiological synchrony and support what we have called pedagogical resonance: the emergence of a shared, coherent pattern across brains and bodies that scaffolds learning, meaning-making, and group cohesion.
3. Affective and autonomic co-regulation
  Coherence is not purely cognitive; it is deeply linked to the regulation of affect and autonomic states. EMI–EDU highlights the capacity of educators to act as co-regulators of emotional and physiological arousal, using tone of voice, posture, facial expressions, narrative framing, and explicit validation of emotions. By reducing “noise” generated by anxiety, shame, or threat, and by increasing signals of safety and recognition, the system can shift from rigid or chaotic patterns toward open, integrative attractor states in which exploration, empathy, and reflective thinking become possible.
4. Pointer and environment design
  EMI-EDU conceptualises the physical and symbolic environment as a set of pointers that guide the system toward particular regions of its state space. The notion is adapted from Levin’s account of Platonic space, in which biological systems navigate structured landscapes of possible patterns guided by setpoints accessed through interface structures (Levin, 2022; Levin and Martyniuk, 2018). Within EMI-EDU, pedagogical practices, classroom artefacts, rituals, and environmental configurations function as such pointers: they make specific cognitive, affective, and relational patterns more accessible to learners by shaping the local geometry of the attractor landscape. Concretely, this includes classroom layout, lighting, sound, colours, and material artefacts, as well as shared symbols, narratives, and rituals. These pointers can be intentionally configured to make certain trajectories more accessible than others, for instance, cooperation rather than competition, curiosity rather than withdrawal. From this perspective, the educational environment is not a neutral backdrop but an active component of the resonant field, and the teacher’s role partially reframes as that of an interface designer who shapes the conditions under which new attractors can stabilise. Assessment, in turn, can be understood as the measurement of error-minimisation relative to desired developmental setpoints.

These four domains provide educators with concrete handles for influencing the resonant configuration of the system. They do not replace content or curricula, but they frame *how* content is delivered and *how* learning relationships are structured, with the explicit goal of enhancing coherence across levels.

### 5.3 The EMI–EDU protocol: sense, tune, stabilise

To make these principles operational, EMI–EDU proposes a simple three-step protocol that can be applied at different scales (from the micro-interaction to the whole-school level):

1 Sense the field
    The first step is to develop the ability to *sense* the current configuration of the educational field. This involves systematic observation of:

- behavioural indicators (engagement, turn-taking, posture, movement patterns);
- emotional climate (dominant affects, signs of safety or threat, relational trust);
- where feasible, physiological and neural markers (e.g., heart rate variability, hyperscanning indices, electromyographic cues). These observations are interpreted in terms of attractor landscapes and evolutionary entropy: is the system rigid and over-organised, chaotic and fragmented, or in a metastable regime that combines differentiation and integration?

2 Tune the Field
    Based on this diagnosis, educators tune the field by deploying one or more of the levers described above. This may involve adjusting temporal rhythms (introducing a pause, a ritual, a brief rhythmic or movement-based activity), modifying embodied interaction (changing position, engaging in shared movement or vocalisation), implementing affective co-regulation strategies, or reconfiguring environmental pointers (rearranging space, introducing symbolic objects, reframing tasks). The goal is to perturb the system sufficiently to enable a transition toward more coherent attractor states, without imposing rigid order or suppressing diversity.
3 Stabilise and encode
    When a more coherent and inclusive configuration emerges, the next step is to stabilise it. Repetition, ritualisation, and explicit metacognitive reflection allow the new pattern to be encoded in the material substrate of the brain and body and, simultaneously, in the relational and institutional fabric of the school. In EMI terms, resonance patterns that are repeatedly instantiated become engraved as informational structures in the system’s mass–energy distribution, altering its future trajectories and increasing its capacity for adaptive, inclusive functioning.

This protocol can be implemented informally in daily practice or formalised in structured programmes and interventions. Importantly, it remains compatible with different pedagogical traditions, providing a biophysical and systemic meta-framework rather than prescribing specific techniques.

Concrete examples. To illustrate how the protocol unfolds in practice, we describe four brief scenarios.

Scenario 1—Primary school, transition between activities. A teacher senses that the class is in a fragmented configuration after a high-arousal break: scattered attention, inconsistent posture, low coordination. Rather than imposing immediate cognitive demand, the teacher introduces a 90-s rhythmic activity (synchronised clapping followed by guided breathing) before transitioning to the next task. The protocol unfolds as: sense (fragmentation), tune (rhythmic embodied synchronisation), stabilise (encoding through the recurrence of the same micro-ritual at each transition over weeks). Measurable signatures over time would include increased behavioural synchrony at transitions and reduced post-transition latency to task engagement.

Scenario 2—Secondary school, conflict in group work. During a collaborative task, a small group becomes locked in a chaotic-defensive configuration with raised voices and disengagement. The teacher’s intervention is not corrective but co-regulatory: lowered prosody, validation of emotional states, brief reframing of the task. The protocol unfolds as: sense (chaotic-defensive attractor), tune (affective and autonomic co-regulation through tone and validation), stabilise (return to task with the group’s experience of having moved through a difficult state, encoding the possibility of recovery). HRV synchrony and turn-taking entropy would be expected to shift toward more integrated patterns.

Scenario 3—University seminar, pointer design. A teacher anticipates that a forthcoming seminar topic risks generating passive reception. Before the session, the teacher reconfigures the room from frontal to circular layout, introduces a shared visual artefact at the centre, and opens with a brief collective question rather than a lecture. The protocol unfolds as: sense (anticipated passive configuration), tune (pointer redesign and opening question as resonance trigger), stabilise (the pattern, if effective, becomes the seminar’s recurrent ritual). Turn-taking entropy and gaze-distribution measures would be expected to differ significantly from frontal-lecture controls (cf. P4 in Section 5.5).

Scenario 4—Recognising a phase transition in progress. A mathematics teacher has observed a student struggling for weeks with algebraic equations, with little incremental progress. Over a few days the student’s performance becomes unstable: he alternates moments of unexpected clarity with elementary errors he had not made before, and his overall accuracy temporarily drops. Rather than reading this regression as failure, the teacher recognises it as the signature of an ongoing reorganisation: the previous cognitive configuration is destabilising, and a critical instability precedes the leap. A few days later the student starts approaching equations in a qualitatively different way, no longer following memorised procedures step by step but perceiving the structural relationships between terms as a unit. Within weeks the task that previously required effortful processing becomes automatic, with reduced bodily tension, faster response times, and the capacity to articulate the underlying logic in his own words rather than reciting definitions. The protocol unfolds as: sense (recognising critical fluctuations and transient U-shaped performance as positive markers rather than regressions), tune (avoiding corrective pressure during the unstable phase, sustaining engagement through low-stakes exploration), stabilise (consolidating the new attractor through repeated application across varied contexts). Behavioural and didactic indicators of the transition complement the neurophysiological signatures discussed in Section 5.5.

### 5.4 Novelty and implications

From this perspective, EMI–EDU can be distinguished from other approaches to neuroeducation and complexity-informed pedagogy in several respects.

First, it offers a bi-functional view of the mind as both *field* and *organism*, explicitly linking phenomenology, neural dynamics, bodily processes, and educational practices through a unified energy–mass–information framework. This allows coherence and resonance to be conceptualised not merely as metaphors, but as phenomena with measurable signatures across scales.

Second, EMI–EDU integrates these signatures into an operational model that identifies concrete levers for intervention and formalises a protocol (sense–tune–stabilise) for inducing and maintaining resonant states. In doing so, it reframes classroom management, curriculum design, and inclusive education as problems of field modulation rather than of individual correction or deficit compensation.

Third, by grounding educational processes in evolutionary entropy, EMI–EDU provides a way to evaluate the long-term effects of pedagogical interventions on the system’s capacity for complex, integrated behaviour. Coherent, resonant configurations are not valued only because they improve immediate performance or well-being, but because they orient the developmental trajectory toward greater differentiation, integration, and systemic inclusion.

**Figure 1:**
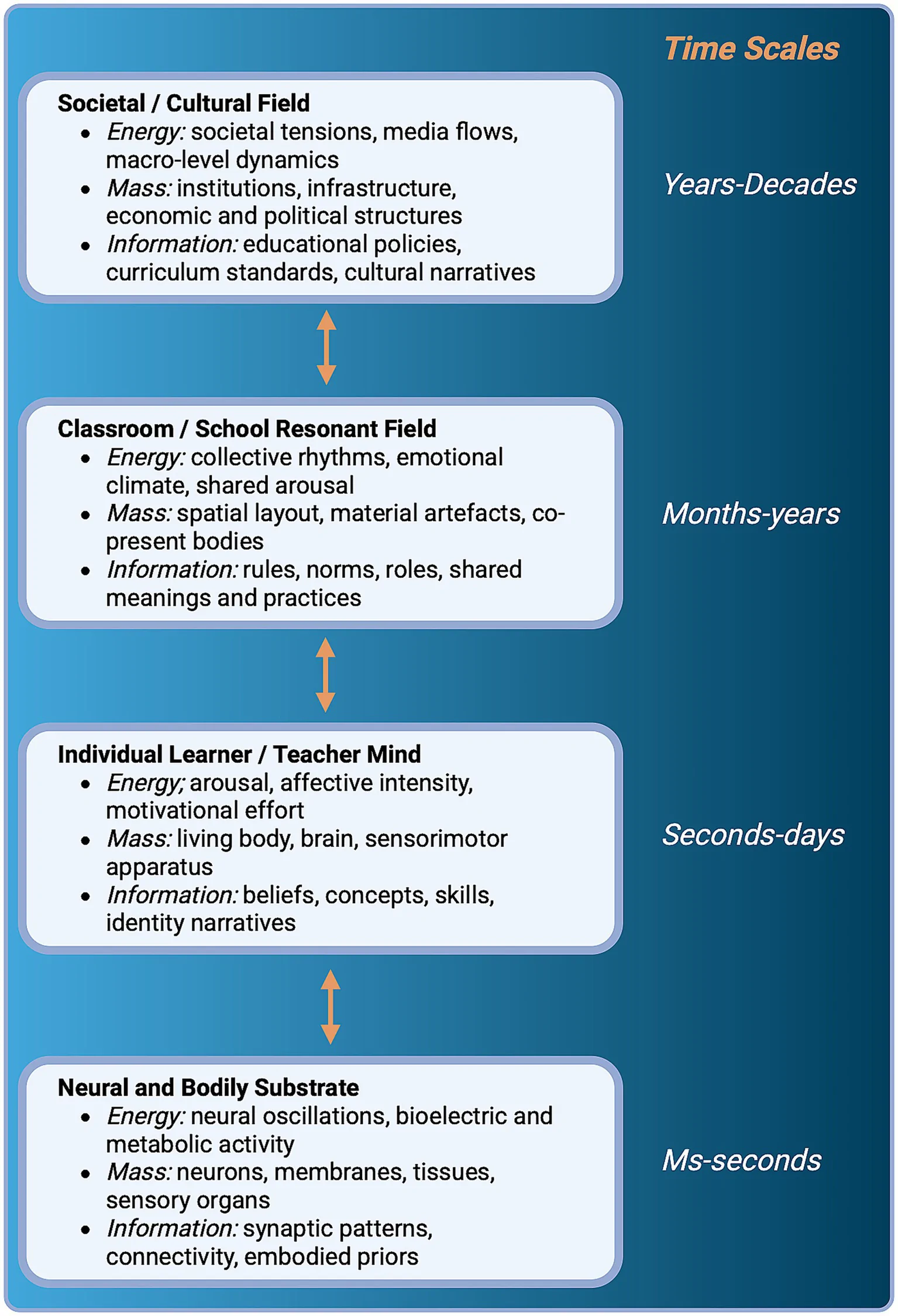
Multiscale organisation of the EMI–EDU framework. The EMI–EDU model describes educational processes as emerging from resonant interactions across four coupled levels: The neural and bodily substrate, the individual learner/teacher mind, the classroom/school resonant field, and the broader societal and cultural field. At each level, energy, mass, and information assume specific forms (e.g., neural oscillations and synaptic patterns at the neural level; affect, embodiment, and identity at the individual level; rhythms, spatial layout, and shared practices at the classroom level; policies and cultural narratives at the societal level). Bidirectional flows of energy–mass–information couple these levels, so that coherence and resonance can propagate across scales, shaping learning trajectories and systemic inclusion.

Finally, the model invites new lines of empirical research. Hyperscanning, autonomic measures, behavioural and phenomenological data can be combined to study how specific temporal, embodied, affective, and environmental manipulations reshape the attractor landscape of classrooms and schools. In this way, EMI–EDU opens a path toward a resonance-based pedagogy that is at once theoretically grounded, practically actionable, and empirically testable (Figure 1).

### 5.5 Empirical predictions

The framework articulated in the previous sections generates a set of falsifiable predictions that distinguish EMI-EDU from purely descriptive accounts of educational complexity. These predictions specify direction, magnitude anchored to existing benchmark literature, and the empirical conditions under which the framework would be disconfirmed. They are intended to operationalise the heuristic constructs introduced in earlier sections (the classroom as a resonant field, the sense–tune–stabilise protocol, pointer design) into observable and measurable claims, in line with the epistemological registers articulated in §2.1. Table 2 summarises four primary predictions; a longer-term research programme addressing developmental trajectories is outlined in §6.

### 5.6 Positioning relative to existing frameworks

EMI-EDU emerges in dialogue with several established traditions in cognitive science and education. We address the most directly comparable frameworks below.

Free Energy Principle and Active Inference (Friston, 2010; Clark, 2013). EMI-EDU and FEP share a probabilistic-dynamical view of cognition and a rejection of linear input–output models of learning. The two frameworks operate, however, at complementary levels of description: FEP characterises adaptive behaviour in information-theoretic terms (variational free energy, prediction error), while EMI-EDU specifies the biophysical substrates, oscillatory regimes, autonomic dynamics, lipid-membrane organisation, through which educationally relevant dynamics become measurable (Cavaglià and Tuszynski, 2026; Firaux et al., 2026). EMI-EDU also differs from FEP in being articulated from the outset as a trans-individual framework, in which inter-brain synchrony and shared phenomenological fields are constitutive rather than derivative (Friston and Frith, 2015), a feature germane to educational settings, where learning is intrinsically relational.

4E Cognition (Newen et al., 2018). EMI-EDU shares with the 4E framework the rejection of brain-bound computationalism and the emphasis on body and environment as constitutive of cognition. Where 4E offers a phenomenological vocabulary, EMI-EDU adds a biophysical and predictive layer, articulating how the four “Es” couple across scales through measurable energetic and informational dynamics (Section 5.5).

Embodied Education and the Italian pedagogical tradition (Frauenfelder Elisa, 1997; Frauenfelder et al., 2018; Damiani et al., 2021; Sibilio, 2021; Gomez Paloma and Damiani, 2015). EMI-EDU is a direct continuation of the Embodied Education tradition developed in Italian pedagogical research. Frauenfelder and Santoianni’s notion of bioeducation anticipated, in pedagogical terms, the trans-disciplinary integration that EMI-EDU now articulates in biophysical terms. The “bridge dimensions” between neuroscience, psychoanalysis, and pedagogy (Damiani and Gomez Paloma, 2020) acquire, within EMI-EDU, specifiable energetic-informational referents and corresponding empirical pathways.

Constructivism (Piaget, 1970; Guidano, 1991; Guidano, 1987) and Sociocultural theory (Vygotsky, 1978; Lave and Wenger, 1991). Constructivism and sociocultural approaches frame learning as the active construction of meaning within social and cultural contexts. EMI-EDU does not replace these frameworks but proposes biophysical re-descriptions of their core constructs to be tested empirically: constructivist meaning-making as the stabilisation of attractor configurations through repeated resonant experiences, and the Vygotskian zone of proximal development as the region of the attractor landscape accessible to the learner only through coupling with a more experienced partner.

Synthesis. EMI-EDU’s distinctive contribution is to provide a *trans-scale biophysical grammar* that articulates how phenomena described phenomenologically (4E, Embodied Education), inferentially (FEP), or socially (Sociocultural theory) couple to specifiable biophysical and informational dynamics. The framework’s value is therefore primarily *translational*: it enables claims from different traditions to be expressed in commensurable terms, and it generates predictions (Section 5.5) that test the couplings rather than the individual traditions themselves. Table 3 summarises the relationships.

**Table 3:** EMI-EDU in dialogue with existing frameworks.

Framework	What EMI-EDU shares	What EMI-EDU adds
FEP / Active Inference (Friston, Clark)	Probabilistic-dynamical view of cognition; rejection of linear input–output models	Specification of biophysical substrates; trans-individual scope as constitutive rather than derivative
4E Cognition (Newen et al.)	Rejection of brain-bound computationalism; body and environment as constitutive	Cross-scale coupling through energetic and informational dynamics; falsifiable predictions
Embodied Education (Frauenfelder, Damiani, Sibilio, Gomez Paloma)	Co-evolution of biological and cultural dimensions; body as mediator of cognition	Biophysical grammar for ‘bridge dimensions’; empirical pathways for phenomenologically articulated constructs
Constructivism (Piaget, Guidano)	Learning as active construction of meaning	Proposed re-description of meaning-making as attractor stabilisation
Sociocultural theory (Vygotsky, Lave & Wenger)	Meaning-making within social and cultural contexts	Proposed re-description of ZPD as accessible region of coupled attractor landscape

## 6 Conclusion and perspectives

In this article we have deliberately adopted a theoretical and conceptual stance: EMI-EDU is presented here as a hypothesis-generating framework rather than as the report of an empirical study. Future work will need to operationalise and test the model in real educational settings, combining behavioural and phenomenological data with physiological and neural measures such as inter-brain hyperscanning, heart rate variability (HRV), and other indices of autonomic regulation. In particular, experimental and field studies could investigate how specific EMI-EDU intervention protocols, including temporal and rhythmic structuring, embodied synchronisation, and affective co-regulation, reshape the attractor landscape of classrooms, increase coherence across scales, and ultimately support more inclusive and adaptive learning trajectories.

The framework articulated here is conceived as a contribution to long-standing pedagogical traditions: from Frauenfelder’s bioeducation to Embodied Education, and back to preventive and scientific pedagogies such as those of Don Bosco and Montessori, in which growth is fostered through authentic, embodied, and contextual experiences rather than through repression or artificial interventions. EMI-EDU does not stand above or beyond these traditions; it seeks to support and valorise their practice by offering a biophysical and systemic grammar commensurable with what pedagogical knowledge has long held as its core insight, that education is a delicate, formative work on the developing person. Resonance, attractor dynamics, and evolutionary entropy translate this insight into a view of inclusive education that recognises how environmental and contextual barriers can constrain human potential, and that frames education as a process of self-organisation toward higher levels of complexity, integration, and flourishing (Damiani, 2017). Of course, principles cannot be transposed in a linear or simplistic manner from one disciplinary area to another: the value of EMI-EDU lies precisely in providing a vocabulary that respects the autonomy of pedagogical reflection while opening it to commensurable empirical investigation.

The EMI model is grounded in the idea of the mind as a mental field that does not end at the boundaries of the body, but extends in functional and resonant continuity with the environmental field. Within this perspective, physical, cultural, social, and relational aspects are integrated through an “evolutionary role” (epigenetics), becoming constitutive elements of the mind itself.

The paradigm shift implied by EMI-EDU invites us to see schools not as static repositories of knowledge, but as living systems in which individual and collective trajectories evolve through meaningful perturbations and careful, attuned guidance. Learning is no longer understood as mere input/output processing, but as the continuous adjustment of an open, self-organising system toward greater integration, awareness, and adaptivity. By grounding pedagogy in the physical and informational principles that govern complex systems, including phase transitions, attractor dynamics, and evolutionary entropy, EMI-EDU offers a scientifically grounded yet deeply humanistic platform that gives empirical depth to what skilled educators already do: tuning, accompanying, and helping the learner unfold.

Understanding mental functioning and neurodiversity through the EMI perspective offers two complementary contributions. First, it expands our knowledge of the limits and obstacles to mental functioning while, more importantly, identifying new levers and resources for intervention. Second, it encourages a broader view of relational and environmental contexts, recognising the diverse challenges and opportunities they present and supporting the redesign of educational and formative practices that promote co-development and flourishing within an inclusive framework. Expanding the field of action in this way naturally increases the range of possible resources and avenues for intervention.

Looking ahead, our research will focus on exploring this transdisciplinary field, which we have defined as “EMI-EDU,” from both theoretical and applied perspectives, to promote a pedagogy of resonance, plasticity, and systemic inclusion that recognises and values the intrinsic complexity of the human subject and of contemporary educational environments. Starting from the “extended mental field” model and the principle of synchronisation outlined above, we will further investigate the role of the teacher as one who attunes and accompanies, supporting the emancipatory and empowering self-organisation of the teacher-student dyad and of the class-school system in close connection with the development of their relational capacities. In this light, education emerges not as the transmission of contents but as a formative act in the deepest sense: relation, and therefore education, shapes the very matter of which we are made.

## Data Availability

The original contributions presented in the study are included in the article/supplementary material, further inquiries can be directed to the corresponding authors.
